# Application of Artificial Intelligence in Gastrointestinal Disease

**DOI:** 10.3390/diagnostics16050723

**Published:** 2026-02-28

**Authors:** Kai Zhao

**Affiliations:** School of Communication and Information Engineering, Shanghai University, Shanghai 200444, China; kz@kaizhao.net

## 1. Introduction

Gastrointestinal (GI) diseases represent a diverse group of conditions affecting individuals of all ages, with increasing global prevalence and a strong association with cancer-related morbidity and mortality. Limited medical resources, however, pose substantial barriers to effective early diagnosis [1]. Artificial intelligence (AI) and especially machine learning have emerged as transformative forces in gastroenterology, offering new approaches for diagnosing, treating, and managing gastrointestinal (GI) diseases [2,3,4]. In recent years, AI-driven tools—ranging from computer vision algorithms for endoscopic image analysis to predictive models for patient outcomes—have demonstrated the potential to enhance clinical accuracy and efficiency [5,6,7,8]. In a study by Kim et al. [9], AI-assisted colonoscopy video integration was shown to enhance detection sensitivity, especially in real-world settings. These advances underscore a paradigm shift in GI healthcare: leveraging data and algorithms to support physicians in making earlier, more accurate diagnoses and in personalizing patient care. AI is also being applied to functional disorders: Lundervold et al. [10] used machine learning on brain MRI and cognitive data to identify IBS patients with 93% sensitivity (22% specificity), highlighting the role of brain–gut axis dysregulation.

This Special Issue, titled “Application of Artificial Intelligence in Gastrointestinal Disease,” presents eight papers that collectively illustrate the breadth of current AI applications in the field. The contributions span from image-based diagnosis (in endoscopy, pathology, and radiology) to outcome prediction and even patient-centered communication, reflecting the multifaceted impact of AI on GI disease management.

## 2. Contributions to This Special Issue

Several papers in this issue focus on improving the detection of GI lesions through AI-augmented endoscopy. Building on prior clinical evidence demonstrating the effectiveness of real-time AI-assisted polyp detection and optical diagnosis during colonoscopy [11], the studies included in this Special Issue further explore the clinical utility of AI-assisted colonoscopy in routine clinical practice and across different endoscopist experience levels. In particular, Kim et al. [9] developed a polyp detection model using semi-automatically annotated colonoscopy videos, which achieved 90.6% sensitivity and 96.0% specificity—outperforming models trained on public datasets. Chitca et al. [12] provided a comprehensive review of AI’s integration into colonoscopy, discussing clinical performance, implementation challenges, and its potential as a training tool.

Beyond the colon, AI is also transforming upper GI endoscopy. In this issue, Lee et al. [13] report on an AI system for detecting gastric neoplasms in endoscopic images. Their validation study in an upper GI setting showed that the algorithm could accurately identify gastric lesions with an overall accuracy of about 88–94%. Perhaps more critically, when endoscopists—especially those with less experience—used AI assistance, their diagnostic sensitivity and specificity markedly improved. This finding suggests that AI can serve as a valuable “second pair of eyes” during endoscopic examinations, helping catch subtler lesions that might be missed by human observers and thus acting as a real-time educational aid for trainees.

Other contributions extend the application of AI to GI pathology and prognosis. Aneiros-Fernández et al. [14] developed a deep learning model for automated histopathological analysis, targeting the detection of *Helicobacter pylori* in gastric biopsy samples. Using Warthin–tarry-stained slides, their approach achieved over 92% precision and 98% recall in identifying *H. pylori* bacteria. By drastically reducing the time required to screen biopsy images, this AI tool can assist pathologists in rapidly and consistently detecting infections that are risk factors for gastric cancer. Complementing histopathological analysis, Yepuri [15] illustrated how an AI-driven tissue system pathology test (TSP-9) can stratify Barrett’s esophagus patients by individual risk of progression to esophageal adenocarcinoma, enabling personalized surveillance or early intervention. In the realm of oncologic imaging, Park et al. [16] demonstrated that a radiomics-based machine learning model using routine abdominopelvic CT scans could predict occult bone marrow metastases in gastric cancer patients with high accuracy (AUC = 0.96), even when lesions were invisible on conventional CT review. Beyond structural and oncologic assessment, AI has also been applied to functional gastrointestinal disorders. Lundervold et al. [10] combined brain morphometric features with cognitive measures to predict irritable bowel syndrome using machine learning, highlighting the potential of AI to capture brain–gut interactions that are difficult to assess through conventional clinical evaluation alone. Such prognostic AI models could aid emergency and critical care teams by identifying high-risk patients who might benefit from more intensive monitoring or early interventions.

Finally, Anand et al. [17] tested ChatGPT-generated summaries of perianal fistula MRI findings. The AI-enhanced reports improved readability and patient comprehension, though clinicians noted occasional factual inaccuracies, underscoring the need for human oversight in patient-facing applications.

Collectively, the papers in this Special Issue exemplify the diverse ways in which AI is being harnessed to advance gastrointestinal disease diagnostics and care. From enhancing the detection of neoplastic lesions in endoscopic imaging to accelerating pathological analysis and improving patient literacy regarding their conditions, these studies demonstrate both the current capabilities and the future promise of AI in gastroenterology.

## 3. Future Outlook

The advances highlighted in this Special Issue underscore both the promise and the profound responsibilities accompanying AI adoption in gastroenterology. While AI tools—such as multi-lesion classification models—are increasingly embedded in clinical workflows, their implementation raises ethical concerns that extend well beyond diagnostic accuracy to include data privacy, algorithmic bias, health equity, patient autonomy, and the integrity of the clinician–patient relationship. These systems rely on vast, heterogeneous datasets encompassing imaging, clinical histories, and demographic information to achieve high performance. Even with standard anonymization practices, the risk of re-identification persists as datasets grow in scale and detail, necessitating stringent data security measures and strict compliance with regulations such as the General Data Protection Regulation (GDPR) [18,19]. Robust ethical and regulatory frameworks are thus essential not only to prevent misuse but also to build public trust and ensure equitable access to AI-enhanced care.

Moving forward, several priorities emerge for future research. Chief among them is the need for large-scale, multicenter prospective validation to confirm that AI models generalize across diverse populations and real-world settings—addressing current limitations rooted in retrospective evidence [20]. Equally important is the development of human-centered deployment strategies that augment, rather than replace, clinical judgment. As conversational AI begins to support patient communication, human oversight remains critical to mitigate risks of misinformation and maintain safety. Evolving regulatory standards must prioritize transparency, fairness, and clinical efficacy, while interdisciplinary efforts should focus on hybrid approaches that integrate AI-driven imaging with genomic and clinical data to enable truly personalized GI care—all grounded in ethical implementation and responsible innovation. 
